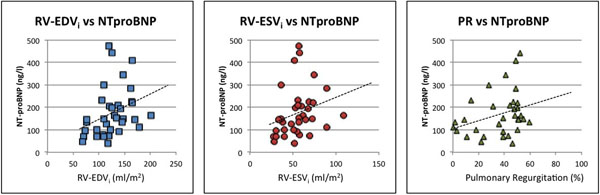# NT-proBNP as a biomarker of right ventricular dilatation and pulmonary regurgitation in Tetralogy of Fallot

**DOI:** 10.1186/1532-429X-17-S1-Q100

**Published:** 2015-02-03

**Authors:** Israel Valverde, Annalisa Paolino, Maria Pilar Serrano Gotarredona, Silvia Navarro, Nieves Romero, Joaquin Fernandez-Cruz

**Affiliations:** 1 Paediatric Cardiology, Hospital Virgen del Rocio, Seville, Spain; 2 Cardiovascular Pathology Unit, Institute of Biomedicine of Seville (IBIS), Seville, Spain; 3 Radiology, Hospital Virgen del Rocio, Seville, Spain; 4 Cardiology, Hospital Virgen del Rocio, Seville, Spain

## Background

After surgical correction of Tetralogy of Fallot (TOF), residual pulmonary regurgitation (PR), right ventricular (RV) dilatation and RV failure have been associated with adverse clinical events and are considered criteria for pulmonary valve replacement. Cardiovascular magnetic resonance imaging (CMR) is the gold-standard for evaluation of these parameters. However it is an expensive technique which is not readily available in all hospitals and outpatient clinics. NT-proBNP is a biomarker which may overcome these CMR limitations and might be used as a cost-effective biomarker for monitoring patients with TF.

The aim of this study is to evaluate whether NT-proBNP plasma levels may help as a biomarker for monitoring ventricular dilatation, pulmonary valve regurgitation and heart failure in patients with repaired TF

## Methods

Single-centre observational prospective study including 40 patients (14.3±6.7 years, mean±standard deviation) with corrected TOF referred to our MRI unit. Data collection included: Clinical Parameters (electrocardiogram, chest X-ray, NYHA scale, time since last surgery), Biochemistry (NT-proBNP levels) and CMR (ventricular volumetry assessment by 2D-SSFP sequence (TR/TE=3.0/1.5ms, flip angle=60°, resolution 1.7x1.7x8mm, SENSE=2) and aortic and pulmonary flow assessment by 2D-phase contrast flow (TR/TE=4.4/2.4ms, flip angle=10°, resolution 1.5x1.5x8mm, averages=2).

## Results

Mean time since last surgery was 13.1±6.3 years. Most patients were asymptomatic (median NYHA scale 1), with QRS duration of 125±19 ms and a cardiothoracic ratio of 0.57±0.1. The mean NT-proBNP was 175±109 ug/ml. CMR analysis revealed a dilated indexed RV-end-diastolic-volume (RV-EDVi) of 125±32 ml/m2 and RV-end-systolic-volume (RV-ESV_i_) of 55±19ml/m2, moderate to severe PR-fraction of 36±17% and good biventricular ejection fraction (RV-EF 56±7%, LV-EF 60±7%).

The statistical analysis showed a statistically significant correlation between the NT-proBNP and RV dilatation for both the RV-EDV_i_ (Pearson 0.54, p<0.01) and RV-ESV_i_ (Pearson 0.52, p<0.01) and also with the PR fraction (Pearson 0.26, p<0.01). There was no statistically significance found between NT-proBNP and RV-EF (Pearson=0.26), LV-EF (Pearson 0.24) or any clinical parameters.

The receiver operating curve analysis evidenced that a NT-proBNP cut-off value above 145 pg/ml predicts the presence RV-EDV_i_ dilatation over over percentile 95 [1] (Sensitivity 71%, specificity 100%), RV-ESV_i_ dilatation over percentile 95^1^ (Sensitivity 88%, Specificity 89%) and severe PR>40% (Sensitivity 72%, Specificity 73%).

## Conclusions

In patients with surgically corrected TOF, NT-proBNP levels correlate with RV dilatation and the degree of pulmonary regurgitation. Ambulatory determination of NT-proBNP might be an easy, readily available and cost-effective alternative for follow-up evaluation of these patients and may help decide when to request an MRI study for follow-up evaluation

## Funding

N/A.

**Figure 1:**